# Proline concentrations in seedlings of woody plants change with drought stress duration and are mediated by seed characteristics: a meta-analysis

**DOI:** 10.1038/s41598-023-40694-5

**Published:** 2023-09-13

**Authors:** Joanna Kijowska-Oberc, Łukasz Dylewski, Ewelina Ratajczak

**Affiliations:** 1 Institute of Dendrology, Polish Academy of Sciences, Parkowa 5, 62-035 Kórnik, Poland; 2 Department of Zoology, Poznań University of Life Sciences, Wojska Polskiego 71C, 60-625 Poznań, Poland

**Keywords:** Forest ecology, Plant cell biology, Plant physiology, Plant stress responses, Drought, Biochemistry, Ecology

## Abstract

Proline accumulation represents one of mechanisms used by plants to prevent the adverse consequences of water stress. The effects of increased proline levels in response to drought differ among species. Trees are exposed to the long-term effects of climate change. The reproductive success of species in a specific environment depends on the functional trait of tree seeds. We conducted a meta-analysis to evaluate the effects of drought stress on the proline concentrations in seedling leaf tissues of woody plant species and their relationships to drought duration, seed mass, seed category and coniferous/deciduous classification. Drought duration exhibited a nonlinear effect on proline accumulations. The drought effect on proline accumulations is greater for deciduous than for coniferous species and is higher for orthodox seed species than for recalcitrant. The seedlings of large-seeded species showed greater effect sizes than those of small-seeded species. Our results suggest that there is an optimum level at which proline accumulations under the influence of drought are the highest. A link between seed functional traits, as well as the coniferous/deciduous classification, and proline concentrations in tree seedlings during water stress were determined for the first time. Proline may help to identify high-quality seeds of trees used for reforestation.

## Introduction

Plants in natural conditions are constantly exposed to various types of environmental stressors (e.g., heat, drought, salinity, and heavy metals)^1–4^. These factors cause structural and functional changes that adversely affect the growth, development or productivity of plants^5–7^. Drought is one of the most serious environmental stressors that disrupts the physiological processes associated with plant development. The main consequences of drought consist of damage to DNA, enzymes and cell membrane structures, which cause disruption of cell functions^8,9^. To limit the results of stressor activity, plants have developed physiological^10–13^, phenological^14–16^ and morphological^17–20^ adaptations.

Proline is a protective molecule that accumulates in plants during drought stress. The accumulation of proline is one of the mechanisms that prevent stress conditions^21^. Proline significantly contributes to osmoregulation during the active growth period of seedlings, and its contribution is greater than for other osmolytes^22^. Some evidence has shown that proline levels increase during times of water stress that are experienced by young tree individuals^23,24^. This cyclic amino acid has been a research object for a long time because it acts not only as an osmolyte that reduces water loss^25^ but also affects the vitality of tree offspring, which results from the metabolic activity of cells that are responsible for proper plant development^26^ and adaptations to changing environmental conditions^27,28^. Proline is an important component of many proteins; therefore, it improves the stability of proteins and the integrity of cell membranes, which enables the proper functioning of physiological processes^29^.

Seed functional traits play an important role in explaining plant success with respect to biotic (e.g., seed predation) and abiotic (e.g., water limitation) factors. Seed size is recognized as a fundamental trait for understanding the functioning of plant communities^30,31^ and is linked with other plant ecological factors^32–35^. For example, large-seeded species are associated with higher recruitment survival rates^36–38^ (but see^39^) and provide an advantage for seedling establishment in dry conditions. Moreover, seedlings from large-seeded species had higher emergence and survival percentages than seedlings from small-seeded species in dry soil conditions in glasshouse experiments^32^. One of the integral parts of plant regeneration ecology functional traits is the ability of seeds to survive desiccation^40^. Previous studies have indicated that small seeds, which for a given seed structure, are likely to dry out faster than large seeds^40,41^, and many species, mostly orthodox ones, have small seeds that persist in soil seed banks  ^42,43^. It was observed that seed survival rates under drought conditions increased with seed weights^44–46^. Some evidence suggests that proline concentrations are associated with seed storage behavior (hereafter the seed category). Seeds are broadly classified as ‘recalcitrant’ and ‘orthodox’ based on their desiccation tolerance. Desiccation tolerance has often been considered to be a qualitative feature of species, with seeds either perishing or surviving after experiencing a certain level of drying^47^. Desiccation tolerance (DT) is the ability of an organism to dry to equilibrium with moderately dry air (e.g., 50–70% RH at 20–30 °C) and then resume normal functioning when rehydrated^48^. Orthodox, desiccation-tolerant seeds are capable of tolerating desiccation to low (generally < 7%) moisture contents with little effect on viability^49^. In contrast, recalcitrant^50^, hereafter desiccation-sensitive seeds, are killed by desiccation to water contents as high as 20–30% and are therefore difficult to store except for the short term^51^. The viability of seeds is a manifestation of their tolerance to desiccation, as they are characterized by seeds that are better protected against damage in dry conditions. It is noteworthy that for many species, the limiting values of the water contents in orthodox seeds and for the onset of near-immediate mortality in resistant seeds are very similar, which range from 0.20 to 0.3 g H_2_O g^−1^ DW^52^. Climate change, which affects the loss of seed viability, may have long-term consequences by reducing species dispersal and causing the acquisition of new habitats^53^. It is predicted that increases in drought intensity will have detrimental effects on seed production in deciduous tree species^54^. Conifers are generally characterized by higher drought tolerance, which is a result of their lower stomatal sensitivity to vapor pressure deficits and lower hydraulic conductivities compared to angiosperms^55^, and thus, the physiological strategies that are activated due to water stress conditions also differ between coniferous and deciduous plants^56^. It was observed that the growth of deciduous tree species was more sensitive to low water availability than the growth of coniferous species; as opposed to conifers, deciduous trees decreased the vascular tissues in their leaves and accumulated proline more intensively^57^.

Despite these observations, there is a lack of research that correlates fundamental seed traits with the physiological markers of drought stress tolerance, such as proline. Studies of the influence of proline on the ability to withstand environmental stresses in tree species have generally focused on the proline contents in tissues of developed trees. Although the redox-state regulation system, including the proline metabolism mechanism and its scavenging abilities, are quite well characterized^58^, but there is an incomplete understanding of how changing climate conditions affect the basic molecular mechanisms of tree seed viability. In addition, studies of the impacts of environmental conditions, such as heat or water deficit, on the oxidative stress effects on seeds usually concern herbaceous plants, such as *Arabidopsis*^59^, rice^60^ and maize^61^, rather than studying woody plants. Meanwhile, the research results for short-lived species do not reflect the mechanisms that occur in tree seeds, which are influenced by various external conditions throughout the maturation period, and their parental plants are influenced by external factors for decades or even centuries during their lifetimes.

The problems of global drought-induced stress and mortality in forests as well as in horticulture tree species are currently alarming and unprecedented^62,63^. By using species distribution modeling based on climate variables, a significant decrease in suitable habitat area was predicted for numerous forest-forming tree species, especially deciduous species^53^. Long-lived organisms, such as trees, are particularly vulnerable to environmental changes. Climate change leads to limited soil water availability for trees, while temperatures gradually increase and droughts are increasingly frequent, long-term and occur over increasingly larger areas^64^. The current climatic conditions are shifting from the ecologically optimum conditions for numerous tree species. The ability of plants to survive in unfavorable environmental conditions depends on events that occur during the first developmental phases, namely those in seeds and during the initial growth of seedlings^65^. The course of germination is one of the earliest phenotypes expressed by plants; thus, it usually undergoes natural selection before other traits. Determinations of the specific competitive and climatic conditions in drought-affected areas, where tree mortality will be concentrated, enable forest managers to effectively identify those areas that are more vulnerable to drought and its consequences^66^. The rapidly changing climate affects the selection pressure on tree populations; therefore, it may cause future changes in tree species distributions by reducing seed viability^67^. Our preliminary research showed that the proline contents depend on the changes in thermal and precipitation conditions during seed maturation; therefore, these changes may be used as biochemical indicators to examine the oxidative changes that occur due to seed development and affect seed viability^68^. These results indicate that proline can be a promising marker that will be helpful for selecting high-quality forest reproductive material, which can be stored and used for forestation or reforestation to reduce economic losses in maintained forests^69^. However, there is still a knowledge gap regarding the extent to which global warming stress (e.g., mainly drought stress) affects proline accumulations in tree seeds and seedlings To comprehend how changing climatic conditions impact the molecular mechanisms associated with seed resistance to drought stress, particularly in trees, we conducted a meta-analysis assessing the effect of drought stress on proline concentrations in seedling leaf tissues of woody plant species. We further evaluated whether proline concentrations are mediated by tree type and seed functional traits (e.g., seed sizes and seed categories). We tested the following hypotheses: (1) proline concentrations in seedling leaf tissues increase with drought duration, (2) proline concentrations in seedling leaf tissues of coniferous species differ from those in deciduous species, (3) proline concentrations in seedling leaf tissues increase with seed mass, and (4) proline concentrations in seedling leaf tissues are lower for orthodox species than for those in the recalcitrant seed category.

## Materials and methods

To obtain studies that quantified the drought stress response of woody plant species to proline accumulations in seedling leaf tissues, we searched the Web of Science Core Collection (1945–2021) for peer-reviewed studies by using the search term combination of proline AND seed* AND (drought OR “water stress” OR “water deficit”) AND (tree OR trees OR “woody plant”). We also screened the studies found via this search for relevant citations. From this literature pool, we filtered studies based on the following criteria. The studies used experiments that were performed on wild-type plants, i.e., seeds for the experiments were obtained from individuals growing in their natural range. We focused on studies that examined the proline accumulations in seedling leaves for both deciduous and coniferous tree species. We chose studies in which the durations (in days) that plants were exposed to drought stress were provided. Data from qualifying studies were extracted directly from the text, tables or figures in the publications using Web Plot Digitizer Version 4.0 (https://automeris.io/WebPlotDigitizer/).

We extracted data on the mean proline accumulations in leaves per species, associated measures of variation (SD or SE) and sample sizes for both drought-stressed plant groups (treatment) and well-watered plant groups (control). The exposure durations to drought stress in each experiment were obtained from studies of a given species.

The information about seed masses we took from published databases: the Seed Information Database^70^ and the Botanical Information and Ecology Network Database^71^. For three species, we did not find any information regarding the seed masses. The seed mass (+ 1) values were natural log-transformed for future analysis. Information regarding the seed category for each plant species (e.g., *orthodox*, *intermediate* or *recalcitrant*) was gathered from the Seed Information Database^70^. For 12 species (40% of cases), we found no information regarding the seed category.

Due to the lack of data on seed mass as well as seed categories, we created three databases for the examination times of plants exposed to drought stress and other factors, e.g., (1) plant type (66 data points in database 1), (2) seed masses and plant type (63 data points in database 2) and (3) seed category (40 data points in database 3) that contained information regarding the effects of drought stress on proline accumulations [S1].

### Statistical analysis

To evaluate the effects of drought stress on proline accumulations, we used multilevel random effect meta-analysis models with an REML estimator in the metafor package^72^ using the rma.mv function in R version 4.1.2^73^. We used the log response ratios of the effect sizes to determine the effects of drought stress on proline accumulations: LnRR = log_e_(*X*_t_/*X*_c_)^74^, where *X*_t_ is the proline accumulation in plants exposed to drought stress (e.g., treatment), and *X*_c_ is the proline accumulation in plants that were well watered (e.g., control). Positive LnRR values indicate increased proline accumulations following exposure to drought stress and vice versa. The study sources was used as a random factor in each model to control the covariance of responses from the same study. To test whether the effect sizes varied significantly among studies we ran models with no moderators for each database by using the *Q*_*t*_ statistic. Thesignificant *Q*_*t*_ value based on a χ^2^ distribution reveal that the variances among effect sizes are greater than those expected from sampling errors alone. Next, we built four models that used the moderators as fixed factors. In the first model we tested the overall relationship between the responses and drought durations (using database 1). For reason nonlinear relationship of time of drought duration supported by improvement of the model AICc score (AICc = – 579.61) as calculated with maximum-likelihood estimation [S2] we included a quadratic term in the base model. In the next model, we tested whether the patterns differed by tree type (e.g., coniferous vs. deciduous) by adding this term and its interaction with drought durations. In the third model, we used the same moderators as in model two, which also included the effect of seed mass and the quadratic term of the seed mass term due to a nonlinear relationship (AICc = – 97.88) (using database 2) [S3]. In the fourth model, we examined the effects of drought duration as well as the quadratic term and seed category (e.g., *orthodox* or *recalcitrant*) (using database 3). We decided to remove the intermediate category from the final analysis due to the small sample size (n = 2). For this model, we did not include an interaction term because of the small sample size for the seed category [S4]. The significances of the moderators were assessed by *Q*_*m*_ test statistic based on the χ^2^ distribution. We used predict function in the metafor package to calculated the mean values of the effect sizes for coniferous and deciduous species and orthodox and recalcitrant seed categories, respectively where, the drought duration was set to the mean value across cases.

Our meta-analysis result were checked with the quality established by Koricheva and Gurevitch^75^ for ecological studies. However, we did not find any publication biases confirmed by Egger's regression test for funnel plot asymmetry (z = 1.85, *p* = 0.064; z = 1.82, *p* = 0.069; z = 1.16, *p* = 0.244, respectively, for the three databases (descriptions above). We did not find that the effect sizes varied negatively with publication year for each model (*Q*_*m*_ = 1.60, *p* = 0.206;* Q*_*m*_ = 2.01, *p* = 0.157;* Q*_*m*_ = 0.70, *p* = 0.402) for the three databases. Additionally, we estimated the fail-safe number, or the number of studies that would have to be added to may affect the results of the meta-analysis^76^. These numbers were 309,819, 286,571 and 105,324, respectively, for the three databases, which indicated that the observed results provide a reliable estimate of the true effects based on the criteria in Rosenberg^77^.

## Results

The global literature search located 28 studies that fulfilled the criteria for assessing the effects of drought stress on proline accumulations in seedling leaf tissues. These studies included 35 woody plant species belonging to 18 families, and 8 species were coniferous and 27 species were deciduous.

**Figure 1:**
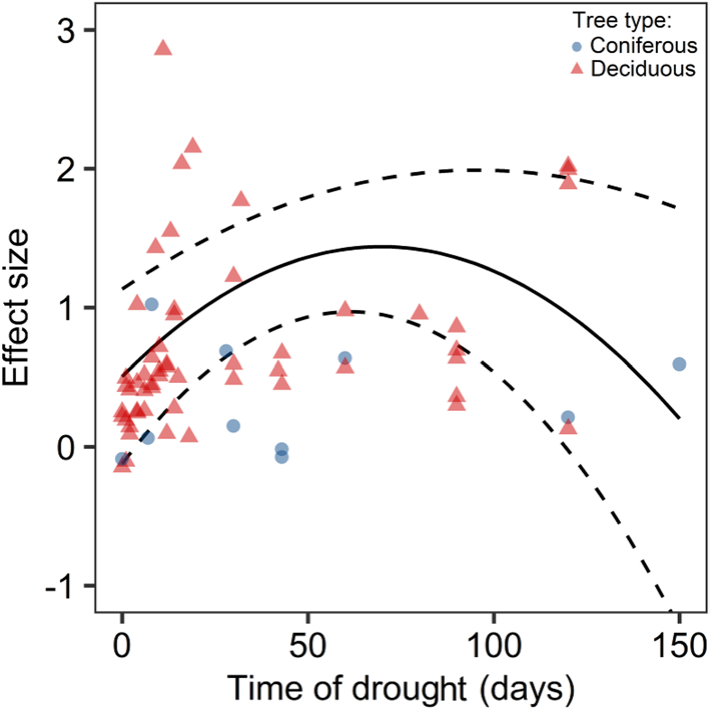
Overall relationship between effects of water stress on proline accumulation in seedlings leaf tissue and duration of drought.The solid line indicates the predicted relationship, and the dotted lines represent the 95% confidence interval. Data points represent a variance in effect size (LnRR).

**Figure 2:**
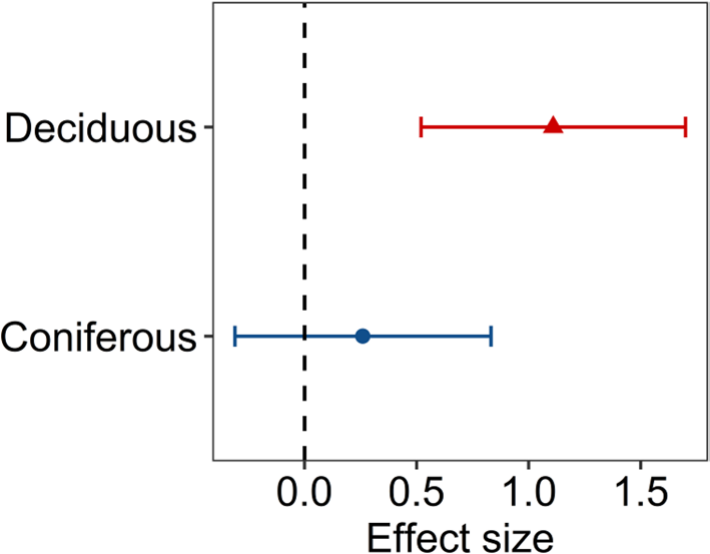
Effect size of water stress on proline accumulation in seedlings leaf tissue between tree type (coniferous vs. deciduous). The points represent mean effect sizes and 95% confidence intervals.

The meta-analysis showed that drought stress significantly increased the proline accumulations in seedling leaves in woody plant species, with a large mean effect size (*g* = 0.79, 95% CI 0.545–1.027) and significant variability among cases (*Q*_*t*_ = 10,069.66, *p* < 0.0001). The first model showed a positive relationship with the number of drought days (*Q*_*m*_ = 19.06, *p* < 0.0001) that was reversed for drought days > 70 days (Fig. 1), as indicated by the significant negative quadratic term (*Q*_*m*_ = 41.55, *p* < 0.0001). The unimodal drought day pattern showed the same patterns as a second model that also accounted for tree type (Table S1). In this model, the interaction terms between drought day andtree type was non-significant (drought day × tree type:* Q*_*m*_ = 0.87, *p* < 0.351; drought day^2^ × tree type: *Q*_*m*_ = 0.04, *p* = 0.847). However, the drought stress effects were significantly greater for deciduous tree species (mean effect size: 1.11, 95% CI 0.521–1.701) than for coniferous species (mean effect size: 0.26, 95% CI − 0.311–0.833, Fig. 2).

**Figure 3:**
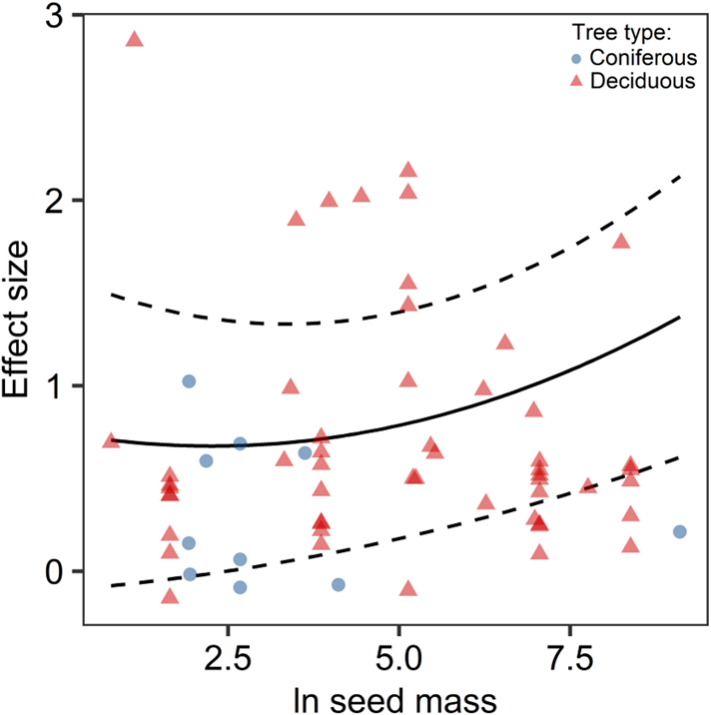
Overall relationship between effects of water stress on proline accumulation in leaf tissue and seed mass,. The solid line indicates the predicted relationship, and the dotted lines represent the 95% confidence interval. Data points represent a variance in effect size (LnRR).

The results from the third model also showed a significant drought day pattern (drought day: *Q*_*m*_ = 43.83, *p* < 0.0001; drought day^2^: *Q*_*m*_ = 14.13, *p* = 0.0002, Table S2). Moreover, the model indicated significant positive relationships with seed mass (*Q*_*m*_ = 133.74, *p* < 0.0001) and the quadratic term of seed mass (*Q*_*m*_ = 100.92, *p* < 0.0001, Fig. 3). The model indicated that the drought stress effects were significantly greater for deciduous tree species than for coniferous species (*Q*_*m*_ = 18.73, *p* < 0.0001). In this model, the drought day relationship also did not vary significantly with tree type (drought day × tree type:* Q*_*m*_ = 0.23, *p* = 0.634; drought day^2^ × tree type: *Q*_*m*_ = 0.84, *p* = 0.360).

**Figure 4:**
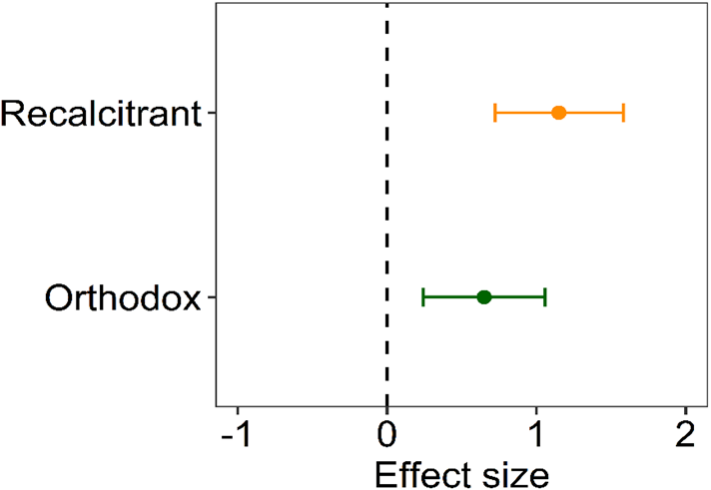
Effect size of water stress on proline accumulation in leaf tissue between seed category (*orthodox* and *recalcitrant*). The points represent mean effect sizes and 95% confidence intervals.

The results from the fourth model indicated a significant drought day pattern (drought day: *Q*_*m*_ = 133.96, *p* < 0.0001; drought day^2^ *Q*_*m*_ = 117.18, *p* < 0.0001, Table S3). Although the drought stress effects were significant between the seed categories (*Q*_*m*_ = 21.26, *p* < 0.0001), the drought stress effects were greater for the recalcitrant seed category (mean effect size: 1.15, 95CI% 0.722–1.581) and orthodox seed category (mean effect size: 0.65, 95CI% 0.243–1.057, Fig. 4).

## Discussion

### The effect of water stress on proline accumulations depends on drought duration and tree type

Proline is one of the most common compatible osmolytes and helps plants maintain homeostasis under drought conditions^78^ by acting as an osmotic adjustment mediator^79^ and a cell redox balance^80^. Its protective mechanisms during water stress involve the stabilization of proteins and antioxidant enzymes, scavenging of ROS and maintenance of redox homeostasis in cells^27^. Our meta-analysis indicated a global pattern of water stress effects on proline accumulations that is dependent on drought duration, with the highest level of proline accumulations occurring at approximately *ca.* 70 days of drought treatment, while this effect was nonlinear (Fig. 1). This pattern suggests that there is an optimum value for which the proline accumulations in seedling leaf tissues under the influence of water stress are high, that is, the prevention mechanism of plants in the face of water deficit, given the full range of times of drought applied in the analyzed studies. This optimum value proceeds to the point where the constant water stress level is probably high enough to generate superfluous ROS that cannot be completely disposed of by the active oxygen scavenging system^81^. The major results of oxidative damage caused by excessive ROS consist of lipid peroxidation, oxidative decomposition of polyunsaturated lipids in the plasma membrane, and disruption of proteins, including the enzymes involved in the antioxidant system, which thereby affects proline metabolism^82^. The structures and functions of cells and tissues are damaged, which consequently leads to death of the organism. Moreover, our data indicate that different types of trees represent different parts of the effect size distribution. The water stress effects on proline accumulations were higher for deciduous than for coniferous tree species (Fig. 2). For the coniferous species, we observed a constant proline concentration value that was independent of drought duration (Fig. 1); however, the interaction term was nonsignificant (*p* > 0.05). This might be due to the divergent evolution of angiosperms and gymnosperms^83^. Conifers are characterized by greater intrinsic water-use efficiency (iWUE) levels than deciduous plants^84^. Although some authors report that under stress conditions, the proline concentrations are usually higher in stress-tolerant than in stress-sensitive plants^85^, our results show that the response in the form of proline accumulations is more intense in deciduous plants, which are considered to be more sensitive to drought conditions. This finding corresponds with the results obtained by Robakowski et al., who noted that a decline in irrigation limited growth and increased the proline concentrations in seedling leaves of deciduous species but not in conifers^57^. Coniferous trees have a cavitation-resistant xylem made of tracheids, while angiosperms, which include all deciduous species included in our meta-analysis, produce tracheids and wide vessels that are characterized by a smaller margin of safety with regard to xylem pressures^55^. Currently, areas are being identified in which the climatic conditions will be optimal in the near future for the most important tree species in forest management in Europe and areas where the emergence of conditions threatening these species is forecasted^86^. Annual precipitation has been indicated as the main variable that explains the current and future distributions of economically important tree species in many Asian countries^63,87^. Hydraulic traits can thus shift species distributions^67^. The proline accumulation levels among seedlings of different tree species may indicate that species suffer more from the emergence of unfavorable environmental conditions, and applying this knowledge to forest management can help predict drought-induced tree mortality^66,68^.

### The effect of water stress on proline accumulations depends on seed mass

Our meta-analysis results show that the trait of seed mass predicted water stress effects on proline accumulations in seedling leaves (Fig. 3). On this basis, it can be concluded that there is a pattern in which species that are characterized by higher seed mass in response to drought accumulate proline more intensively than species with lower seed mass. It can be assumed that survival under drought conditions is connected with increased seed mass, which has already been observed in numerous examples of tree species. In studies focused on the response of *Quercus suber* (L.) to summer drought, the acorn sizes were positively correlated with survival and thus were evaluated as a possible adaptive trait that enhanced stress resistance during further seedling development^46^. This is confirmed by the observations of Reich et al.^45^, who found that seeds appeared to be larger in environments with high drought risks during seedling establishment, and the presence of large seeds in the southern latitudes was explained by the differences in moisture availability along the north–south gradient^45,88^. Summer drought survival increased with seed mass across all families (21 species) that were analyzed by Hallett et al.^44^, and notably, seed mass was correlated with other traits, including root length, which in turn increased the longer-term drought tolerance. Stromberg and Boudell^89^ showed that the seed masses were lower (and less variable) along the channels of long-term river flow sites than in ephemeral locations, which confirms the thesis that seed mass varies depending on the humidity level of the habitat^89^. On this basis, it can be assumed that the features of those seeds that favor withstanding drought conditions consist of both relatively high mass and higher proline accumulations in response to water deficits. Additionally, in this model, the effects of drought stress on proline accumulations were significantly greater for deciduous tree species than for coniferous species. As the effect size is dependent on seed mass and increases with seed mass, this result is predictable. Low seed mass, a short period of juvenile development and short intervals between masting years are typical characteristics of the reproductive strategy of most conifers^90^, while for deciduous trees, there are considerable variations in seed mass.

### The effect of water stress on proline accumulations depends on seed category

According to the results of the meta-analysis, the leaf tissues of seedlings from species in the orthodox seed category accumulate less proline in response to drought stress than those with recalcitrant seeds (Fig. 4). This observation suggests that there is a relationship between seed category and the ability of seedlings to endure drought stress. The seed desiccation tolerance is the ability to counteract water loss (e.g., more than 90% of the total water content) by inhibiting growth and development and then restoring a normal metabolism upon hydration without experiencing tissue damage^51^. Desiccation tolerance in orthodox seeds is acquired as a programmed developmental process and not as a physiological response to the water shoulder^91,92^. Acquisition of desiccation tolerance to low water contents is associated with the presence of min. protective late embryogenesis abundant (LEA) and small heat shock (sHSP) proteins and appropriate levels of sugars. Desiccation-tolerant cells can contain up to 20% of intrinsically disordered proteins (e.g., LEA and sHSP)^93^. LEA proteins have also been detected in cuttings, stems, leaves and roots in response to abiotic stresses, including drought, salinity, heat and cold^94,95^. Orthodox seeds can survive in a dry state thanks to so-called glass structures. During seed maturation, progressive water loss induces the formation of so-called “cytoplasmic glass”, which is an amorphous, sticky matrix that resembles a solid-like state in which the thermal mobility and relaxation rates of molecules are severely slowed down, which thereby contributes to seed protection^96^. Recalcitrant seeds are metabolically active and susceptible to desiccation and also lack the desiccation tolerance mechanisms that allow orthodox seeds to dry out and survive with very low water contents. Another factor that influences the sensitivity to desiccation of these seeds is the lower activity of the antioxidant system compared to that of orthodox seeds^97^.

**Figure 5:**
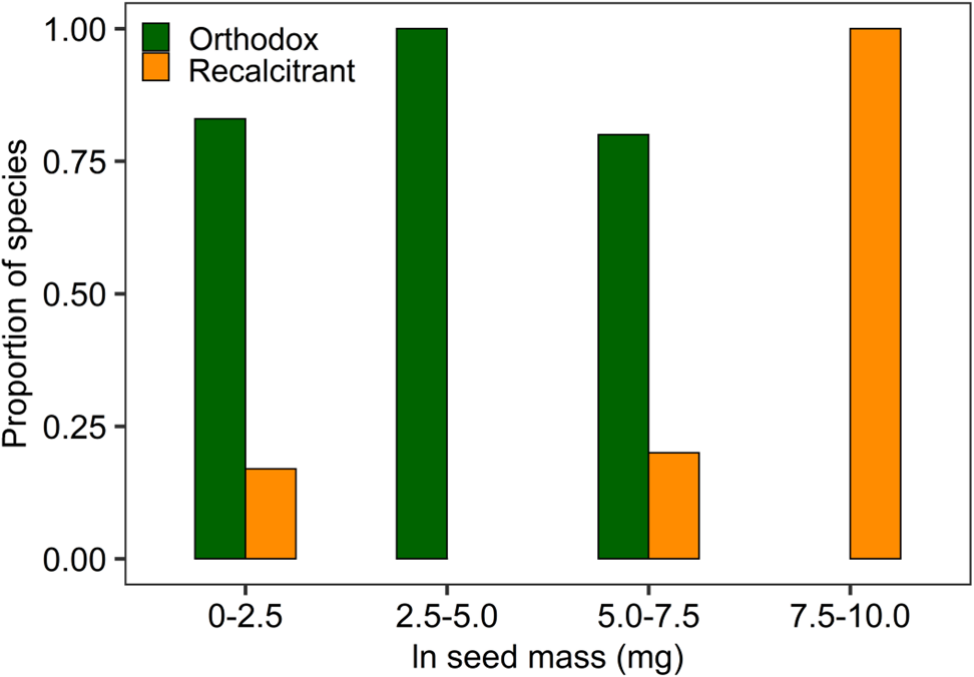
Seed masses distribution for plant species used in meta-analysis of effect of drought stress on seedlings by seed category.

Our study shows that the effect sizes differ among species, whose seeds are assigned to other categories. Seedlings of species with seeds that are more resistant to drought show lower effect sizes in comparison to species with more sensitive seeds (Fig. 4). This new observation is not fully confirmed by the requirements of young trees regarding the environmental conditions during their growth. Among the species included in the meta-analysis, *Quercus robur* (L*.*), a species characterized by recalcitrant seeds, is often found on moist or wet sites and can even survive flooding to a certain extent^98,99^. *Broussonetia papyrifera* (L.) Vent., which also produces recalcitrant seeds and is recognized as a pioneer species in adverse environments because of its adaptable ability^100^. Species that produce seeds that are classified as orthodox are generally found in dry or rocky habitats, and the range of environments that is suitable for their spread is wide, as in the case for *Gleditsia triacanthos* L.^101^ and *Olea europaea* (L.)^102^, while *Picea abies* (L.) H. Karst is an example of an orthodox seed species for which drought resulting from global warming poses a serious risk in the form of rapid reductions in range limits^6,53^. Our results correspond to the seed size mode, where small seeds are mainly categorized as orthodox seeds. The small sample size for this seed category means that we cannot state with confidence that small seeded-species fall mostly in the orthodox category due to the lack of interactions in the model (Fig. 5). Classifying seeds as fully orthodox or recalcitrant is not entirely precise because within each category there is a seed sensitivity gradient, which must be considered when discussing the results. The manner in which the differences between fully orthodox and fully recalcitrant seeds develop in response to drought remains unexplained and requires more research^103^. Despite this, our observations on the relationship between seed category and the biochemical responses of seedlings to drought stress shed new light on plant reproduction ecology and indicate the need to extend research to examine the proline accumulations in seedlings from various tree species.

## Conclusions

In the first global meta-analysis of the effect of drought stress on proline concentrations in seedling leaf tissues in woody plant species, we observed nonlinear relationships with drought durations. We observed a unimodal relationship between drought duration and proline concentrations in seedlings. These findings suggest that as the drought duration increases, proline may have a toxic effect on plants, which causes their decline in approximately 70 days, as our analysis shows. This time span is the optimum duration where the proline accumulations are greatest. Our findings are the first to indicate a link between seed functional traits and proline concentrations in seedlings during drought stress. The water stress effects on proline concentrations were lower in seedlings from small-seeded species as well as those in the orthodox seed category. Moreover, the effects of drought were higher for deciduous than for coniferous tree species.

The adaptations of tree species to climate change and their consequences are one of the most important challenges facing modern forestry. Understanding how drought stress shapes seed viability is very important for protecting the seed resources of forest-forming tree species in the face of climate change. Proline is identified as a fast-acting and unambiguous marker of oxidation changes that occur in tree seedlings. It would be useful to identify the properties of forest reproductive materials collected from regions with different local climatic conditions. It may also be used for activities aimed at preserving the stability of tree populations or species in the face of rapidly changing climatic conditions, e.g., by assisted migration, which consists of placing individuals that are better adapted to the forecasted climate changes in a given area. Minimizing the risk of tree stand stability loss by identifying those populations that are characterized by higher offspring viability may be crucial for the continuity of forest ecosystems.

### Supplementary Information

Supplementary Information 1.

Supplementary Tables.

## Data Availability

All data supporting the findings of this study are available within the paper and within its supplementary materials published online.
