# Author Correction: Proline concentrations in seedlings of woody plants change with drought stress duration and are mediated by seed characteristics: a meta-analysis

**DOI:** 10.1038/s41598-023-43341-1

**Published:** 2023-09-26

**Authors:** Joanna Kijowska‑Oberc, Łukasz Dylewski, Ewelina Ratajczak

**Affiliations:** 1 Institute of Dendrology, Polish Academy of Sciences, Parkowa 5, 62-035 Kórnik, Poland; 2 Department of Zoology, Poznań University of Life Sciences, Wojska Polskiego 71C, 60-625 Poznań, Poland

Correction to: *Scientific Reports* https://doi.org/10.1038/s41598-023-40694-5, published online 13 September 2023

The original version of this Article contained errors in the names of the authors Joanna Kijowska‑Oberc, Łukasz Dylewski & Ewelina Ratajczak, which were incorrectly given as Kijowska‑Oberc Joanna, Dylewski Łukasz & Ratajczak Ewelina.

The original Article has been corrected.

