# Supplementary material for: Proline concentrations in seedlings of woody plants change with drought stress duration and are mediated by seed characteristics: a meta-analysis
Source: Sci Rep. 2023 Sep 13;13:15157. doi: 10.1038/s41598-023-40694-5 (PMC10500006; doi:10.1038/s41598-023-40694-5)
Supplement: Supplementary file 2 — Supplementary Tables. [file 41598_2023_40694_MOESM2_ESM.docx]

**Supplementary materials**

**Proline concentrations in seedlings of woody plants change with drought stress duration and are mediated by seed characteristics: a meta-analysis**

**Joanna Kijowska-Oberc^1*^, Łukasz Dylewski^2^, Ewelina Ratajczak^1^**

^1^Institute of Dendrology, Polish Academy of Sciences, Parkowa 5, 62-035 Kórnik, Poland

^2^ Department of Zoology, Poznań University of Life Sciences, Wojska Polskiego 71C, 60-625 Poznań, Poland

**^*^corresponding author:** joberc@man.poznan.pl

Tables S1-S4

Table S1 List of global literature review included in meta-analysis. Additional details (i.e. plant species, plant type, seed characteristic, time of drougth, proline concentration for control and experimental group) are provided in the Excel spreadsheet (Proline_dataset.xlxs).

| **Sigala JA, Uscola M, Oliet JA, Jacobs DF. 2020.** Drought tolerance and acclimation in Pinus ponderosa seedlings: the influence of nitrogen form. *Tree Physiology* **40**: 1165-1177. |
| --- |
| **Robakowski P, Wyka TP, Kowalkowski W, Barzdajn W, Pers-Kamczyc E, Jankowski A, Politycka B. 2020**. Practical implications of different phenotypic and molecular responses of evergreen conifer and broadleaf deciduous forest tree species to regulated water deficit in a container nursery. *Forests* **11**: 1011. |
| **Aalipour H, Nikbakht A, Etemadi, N, Rejali F, Soleimani M. 2020.** Biochemical response and interactions between arbuscular mycorrhizal fungi and plant growth promoting rhizobacteria during establishment and stimulating growth of Arizona cypress (*Cupressus arizonica* G.) under drought stress. *Scientia Horticulturae* **261**: 108923. |
| **Zhang S, Shao L, Sun Z, Huang Y, Liu N. 2020.** An atmospheric pollutant (inorganic nitrogen) alters the response of evergreen broad-leaved tree species to extreme drought. *Ecotoxicology and Environmental Safety* **187**: 109750. |
| **dos Santos JO, de Oliveira LEM, de Souza T, Lopes GM, Coelho VT, Gomes MP. 2019.** Physiological mechanisms responsible for tolerance to, and recuperation from, drought conditions in four different rubber clones. *Industrial Crops and Products* **141:** 111714. |
| **Tatari M, Jafari A, Shirmardi M, Mohamadi M. 2020.** Using morphological and physiological traits to evaluate drought tolerance of pear populations (*Pyrus* spp.). *International Journal of Fruit Science* **20:** 837-854. |
| **Galeano E, Vasconcelos TS, Novais de Oliveira P, Carrer H. 2019.** Physiological and molecular responses to drought stress in teak (*Tectona grandis* Lf). *PloS one* **14:** e0221571. |
| **Jamnická G, Fleischer P, Konôpková A, Pšidová E , Kučerová J, Kurjak D, Živčák M, Ditmarová L. 2019.** Norway spruce (*Picea abies* L.) provenances use different physiological strategies to cope with water deficit. *Forests* **10**: 651. |
| **Lin J, Zhang R, Hu Y, Song Y, Hänninen H, Wu J. 2019**. Interactive effects of drought and shading on *Torreya grandis* seedlings: physiological and growth responses. *Trees* **33**: 951-961. |
| **Plesa IM, González-Orenga S, Al Hassan M, Sestras AF, Vicente O, Prohens J, Sestras RE, Boscaiu M. 2018**. Effects of drought and salinity on European Larch (*Larix decidua* Mill.) seedlings. *Forests* **9**: 320. |
| **Tariq A, Pan K, Olatunji OA, Graciano C, Li Z, Sun F, Zhang L, Wu X, Chen W, Song D, Huang D, Xue T, Zhang A. 2018**. Phosphorous fertilization alleviates drought effects on *Alnus cremastogyne* by regulating its antioxidant and osmotic potential. *Scientific Reports* **8**: 1-11. |
| **Jafarnia S, Akbarinia M, Hosseinpour B, Modarres Sanavi SAM, Salami SA. 2018**. Effect of drought stress on some growth, morphological, physiological, and biochemical parameters of two different populations of *Quercus brantii*. *Forest-Biogeosciences and Forestry* **11**: 212. |
| **Kebbas S, Benseddik T, Makhlouf H, Fatiha AID. 2018.** Physiological and biochemical behaviour of *Gleditsia triacanthos* L. young seedlings under drought stress conditions. *Notulae Botanicae Horti Agrobotanici Cluj-Napoca* **46**: 585-592. |
| **Merz MA, Donahue RA, Poulson ME. 2017**. Physiological response of Garry oak (*Quercus garryana*) seedlings to drought. *Northwest Science* **91**: 140-159. |
| **Yaish MW. 2015**. Proline accumulation is a general response to abiotic stress in the date palm tree (*Phoenix dactylifera* L.). *Genetics and Molecular Research* **14**: 9943-9950. |
| **Zhang Z, Zhang J, Huang Y. 2014**. Effects of arbuscular mycorrhizal fungi on the drought tolerance of Cyclobalanopsis glauca seedlings under greenhouse conditions. *New Forests* **45**: 545-556. |
| **Zarafshar M, Akbarinia M, Askari H, Hosseini SM, Rahaie M, Struve D, Striker GG. 2014**. Morphological, physiological and biochemical responses to soil water deficit in seedlings of three populations of wild pear (*Pyrus boisseriana*). *Biotechnologie, Agronomie, Société et Environnement; Gembloux* **18**: 353-366. |
| **Filippou P, Bouchagier P, Skotti E, Fotopoulos V. 2014**. Proline and reactive oxygen/nitrogen species metabolism is involved in the tolerant response of the invasive plant species *Ailanthus altissima* to drought and salinity. *Environmental and Experimental Botany* **97**: 1-10. |
| **Wu M, Zhang WH, Ma C, Zhou JY. 2013**. Changes in morphological, physiological, and biochemical responses to different levels of drought stress in Chinese cork oak (*Quercus variabilis* Bl.) seedlings. *Russian Journal of Plant Physiology* **60:** 681-692. |
| **Zhang L, Gao M, Zhang L, Li B, Han M, Alva AK, Ashraf M. 2013**. Role of exogenous glycinebetaine and humic acid in mitigating drought stress-induced adverse effects in *Malus robusta* seedlings. *Turkish Journal of Botany* **37**: 920-929. |
| **Cavatte PC, Oliveira ÁA, Morais LE, Martins SC, Sanglard LM, DaMatta FM. 2012**. Could shading reduce the negative impacts of drought on coffee? A morphophysiological analysis. *Physiologia Plantarum* **144**: 111-122. |
| **Li MR, Li Y, Li HQ, Wu GJ. 2011**. Ectopic expression of FaDREB2 enhances osmotic tolerance in paper mulberry. *Journal of Integrative Plant Biology* **53**: 951-960. |
| **Osório ML, Osório J, Vieira AC, Gonçalves S, Romano A. 2011**. Influence of enhanced temperature on photosynthesis, photooxidative damage, and antioxidant strategies in *Ceratonia siliqua* L. seedlings subjected to water deficit and rewatering. *Photosynthetica* **49**: 3-12. |
| **Arji I, Arzani K. 2004**. Effect of water stress on some biochemical changes in leaf of five olive (*Olea europaea* L.) cultivars. In: Özkaya MT, Lavee S, Ferguson L, eds. *ISHS Acta Horticulturae 791.* *V International Symposium on Olive Growing.* Izmir, Turkey, **791**: 523-526. |
| **Qu X, Wang H, Chen M, Liao J, Yuan J, Niu G. 2019**. Drought stress–induced physiological and metabolic changes in leaves of two oil tea cultivars. *Journal of the American Society for Horticultural Science* **144**: 439-447. |
| **Wang D, Huang G, Duan H, Lei X, Liu W, Wu J, Fan H. 2019**. Effects of drought and nitrogen addition on growth and leaf physiology of *Pinus massoniana* seedlings. *Pakistan Journal of Botany* **51**: 1575-1585. |
| **Yooyongwech S, Phaukinsang N, Cha-um S, Supaibulwatana K. 2013**. Arbuscular mycorrhiza improved growth performance in *Macadamia tetraphylla* L. grown under water deficit stress involves soluble sugar and proline accumulation. Plant Growth Regulation **69**: 285-293. |
| **Zou YN, Wu QS, Huang YM, Ni QD, He XH. 2013**. Mycorrhizal-mediated lower proline accumulation in *Poncirus trifoliata* under water deficit derives from the integration of inhibition of proline synthesis with increase of proline degradation. *PLoS One* **8**: e80568. |

Table S2. Results of multilevel meta-analysis of data obtained from a global literature review evaluating effects of drought stress on proline accumulation in leaf tissue (k=66). The base model tested for an overall relationship with time duration of water drought (drought day), while a separate model also considered the influence of tree type (coniferous vs. deciduous). For tree type terms, only coefficients for deciduous species are shown (coniferous species were the reference category, i.e., β=0).

|  | β | SE | *Q_m_* | *P* |
| --- | --- | --- | --- | --- |
| **Base model** |  |  |  |  |
| drought day | 0.0311 | 0.0013 | 19.06 | <0.0001 |
| drought day^2^ | -0.0002 | 0.0000 | 41.55 | <0.0001 |
| **Model include tree type** |  |  |  |  |
| drought day | 0.0360 | 0.0056 | 41.55 | <0.0001 |
| drought day^2^ | -0.0002 | 0.0001 | 7.62 | 0.0058 |
| tree type | 0.8555 | 0.1960 | 19.06 | <0.0001 |
| drought day × tree type | -0.0053 | 0.0057 | 0.87 | 0.351 |
| drought day^2^ × tree type | -0.0000 | 0.0001 | 0.04 | 0.847 |

Table S3. Results of multilevel meta-analysis of data obtained from a global literature review evaluating effects of drought stress on proline accumulation in leaf tissue (k=63). The model tested effect of time duration of water drought (drought day), seed mass and tree type. For tree type terms, only coefficients for deciduous species are shown (coniferous species were the reference category, i.e., β=0).

|  | β | SE | *Q_m_* | *P* |
| --- | --- | --- | --- | --- |
| seed mass | 0.1519 | 0.0131 | 133.74 | <0.0001 |
| seed mass^2^ | -0.0148 | 0.0015 | 100.92 | <0.0001 |
| drought day | 0.0342 | 0.0052 | 43.83 | <0.0001 |
| drought day^2^ | -0.0002 | 0.0001 | 14.13 | 0.0002 |
| tree type | 0.8227 | 0.1901 | 18.73 | <0.0001 |
| drought day × tree type | -0.0025 | 0.0053 | 0.23 | 0.6342 |
| drought day^2^ × tree type | -0.0000 | 0.0001 | 0.84 | 0.3597 |

Table S4. Results of multilevel meta-analysis of data obtained from a global literature review evaluating effects of drought stress on proline accumulation in leaf tissue (k=38). The model tested effect of time duration of water drought (drought day) and seed category ( *orthodox* and *recalcitrant*). For seed category, coefficient for *recalcitrant* is shown sequentially (*orthodox* was the reference category, i.e., β=0).

|  | β | SE | *Q_m_* | *P* |
| --- | --- | --- | --- | --- |
| drought day | 0.0198 | 0.0017 | 133.96 | <0.0001 |
| drought day^2^ | -0.0002 | 0.0000 | 117.18 | <0.0001 |
| seed type | 0.5014 | 0.1087 | 21.26 | <0.0001 |
